# Characteristics and resource needs in patients with vestibular symptoms: a comparison of patients with symptoms of unknown versus determined origin

**DOI:** 10.1186/s12873-020-00361-8

**Published:** 2020-08-31

**Authors:** Martin Müller, Martina B. Goeldlin, Janika Gaschen, Thomas C. Sauter, Stephanie Stock, Franca Wagner, Aristomenis K. Exadaktylos, Urs Fischer, Roger Kalla, Georgios Mantokoudis

**Affiliations:** 1 Department of Emergency Medicine, Inselspital, University Hospital Bern, University of Bern, Bern, Switzerland; 2 Institute of Health Economics and Clinical Epidemiology, Cologne University Hospital, Cologne, Germany; 3 Department of Neurology, Inselspital, University Hospital Bern, University of Bern, Freiburgstrasse, 3010 Bern, Switzerland; 4 University Institute of Diagnostic and Interventional Neuroradiology, Inselspital, University Hospital Bern, University of Bern, Bern, Switzerland; 5 Children’s Hospital of Aarau, Aarau, Switzerland; 6 Medical Skills Lab, Charité Medical School Berlin, Berlin, Germany; 7 Department of Otorhinolaryngology, Head and Neck Surgery, Inselspital, University Hospital Bern, University of Bern, Bern, Switzerland

**Keywords:** Health resources, Vertigo, Dizziness, Emergency department, Resource allocation, Vestibular

## Abstract

**Background:**

Vestibular symptoms are a frequent reason for presenting at the emergency department (ED). Underlying conditions range in severity from life-threatening to benign, but often remain undiagnosed despite extensive investigations. We aimed to identify clinical characteristics that are associated with ED consultations by patients with vestibular symptoms of unknown origin (VUO) and to quantify the ED resources consumed during the investigations.

**Methods:**

This retrospective one-year, single-centre, cross-sectional study assessed ED consultations with patients whose chief complaint was ‘vestibular symptoms’. Data on risk factors, clinical characteristics, management and ED resources were extracted from the administrative database and medical records. Consultations were grouped according to the discharge diagnosis as either VUO or non-VUO. We determined clinical factors associated with VUO and compared ED resource consumption by the two patient groups using multivariable analysis.

**Results:**

A total of 1599 ED consultations were eligible. Of these, 14.3% (*n* = 229) were consultations with patients with VUO. Clinical characteristics included in the final multivariable model to determine associations with VUO were sensory disorders, aural fullness, improvement at rest, absence of situational provocation, pre-existing neurological conditions, and age < 65 years. Patients with VUO had higher total ED resource consumption in terms of physicians’ work and radiology resources, as a result of more use of computed tomography and magnetic resonance imaging.

**Conclusion:**

One in seven emergency patients with vestibular symptoms is dismissed without a diagnosis. Clinical characteristics of VUO patients are distinct from patients in whom a diagnosis was made in the ED. VUO triggers higher ED resource consumption, which can be justified if appropriately indicated.

## Introduction

Vestibular symptoms are a frequent reason for emergency department (ED) consultations [1, 2]. Dizziness is the third most common major symptom presented by patients in general medical practices. It has a 30% lifetime prevalence [3–5] and accounts for approximately 10 million ambulatory visits every year in the United States (US), about a quarter of which are to the emergency department (ED) [2, 6, 7]. The conditions underlying vestibular symptoms have a broad clinical spectrum ranging in severity from benign to life-threatening [4].

A study in the US estimated the total costs to the ED of consultations due to vestibular symptoms to be about $4 billion per year – corresponding to 4% of the total ED costs [8]. Despite those high costs that reflect extensive diagnostic testing, i.e. use of imaging, many patients with vestibular symptoms are discharged without a diagnosis explaining their symptoms [8–11]. We hypothesize that patients in whom the cause of vestibular symptoms could be determined would have a lower average ED resource consumption than patients with vestibular symptoms of unknown origin (VUO). Some diagnoses can be made without instrumental testing (e.g. benign paroxysmal positional vertigo (BPPV) can be confirmed with a positional manoeuvre), resulting in relatively low consumption of ED resources. In contrast, patients with VUO are more likely to undergo extensive testing in an attempt to find an explanation for their problem. On the other hand, one could hypothesize that diagnostic work-up was more basic in VUO patients, leading to a high risk of missing a life-threatening disorder, ultimately resulting in even higher resource consumption, or harm to the patients.

As appropriate allocation of resources is central to good ED management, identification of factors associated with consultations with patients who were discharged with VUO as well as quantification of ED resources needed by such patients is potentially valuable to ED physicians. Within an interdisciplinary tertiary ED, we aimed to describe the clinical characteristics of patients with VUO, to quantify their consumption of diagnostic resources in the ED and compare this with resource consumption by patients with vestibular symptoms of determined origin (non-VUO).

## Methods

### Study design

This was a retrospective cross-sectional study. It included all consultations with patients whose chief complaint was vestibular symptoms seen at the tertiary ED of the Inselspital, Bern University Hospital, Switzerland, during the one-year period 01/01/2013 to 31/12/2013.

### Eligibility criteria

We included all patients with vestibular symptoms as the main complaint who were referred to our ED during the study period, unless they had withdrawn their general consent for further use of their health-related data for research purposes. Vestibular symptoms were defined according to the Classification of Vestibular Symptoms by the Bárány Society. We did not consider visuo-vestibular symptoms, as a symptom description fitting this definition is lacking in Swiss German language [12]. Vestibular symptoms were considered as the main complaint if they were the reason for the ED visit or if such symptoms were among the first three symptoms recorded in the medical report.

We excluded unconscious or aphasic patients as they were not able to report their symptoms. Patients with pre-existing stable vestibular symptoms or postural symptoms due to paresis or a neuromuscular problem were also excluded. We also excluded consultations with incomplete administrative or resource consumption documentation.

### Data extraction

For this analysis, we merged clinical data from the interdisciplinary database on vertigo and dizziness with data on ED resource use (see Database on ED resource consumption).

#### Interdisciplinary database on vertigo and dizziness

The interdisciplinary database on vertigo and dizziness is a retrospective, single-centre, REDCap database based on ED medical reports on patients with vestibular symptoms who had visited the ED in 2013 [13, 14]. Briefly, we manually screened all ED reports from the year 2013 for descriptions of vestibular symptoms. In cases of uncertainty, two neurootology adjudicators reassessed inclusion, exclusion and coding criteria (RK, GM). Vestibular symptoms and concomitant symptoms were recorded from all visits if the same patients had had multiple ED consultations.

The following variables were extracted and coded manually: i) patients’ demographic characteristics and history: age, sex, vascular risk factors; ii) comorbidities, especially pre-existing neurological, psychiatric and cardiovascular comorbidities, and disorders of the ear, nose or throat; iii) symptoms and signs: symptom quality, triggers, factors leading to improvement, concomitant symptoms; iv) assigned diagnosis.

#### Database on ED resource consumption

At our ED, every staff member is regularly trained for billing purposes. Physicians and nurses, as well as laboratory and radiology staff, document the procedures performed on a patient in accordance with the procedural codes set out in the *Tarmed Suisse catalogue* [15].

The database on ED resource consumption consists of procedural codes that were selected and grouped into the following categories by a committee of acute care nurses and ED physicians, in collaboration with the controller of our ED department: i) ED physician’s, neurologist’s and ENT physician’s work, the sum of physician’s check-up efforts, physician’s administrative and medical report work; ii) nurses’ work, iii) laboratory resources (blood and urine analysis); iv) radiology resources (ultrasound, computed tomography, X-rays, magnetic resonance imaging), v) total work or resources (sum of i–v above).

The monetary value of the procedural codes is recorded in tax points (TP, medical currency; 1 tax point corresponds to roughly 1 US$ depending on the hospital; in our hospital it is 0.93 US$). The codes were exported by an ED controller from the administrative database (OpenText Suite for SAP® Solutions, OpenText Corp., Waterloo, Canada).

#### ED visits by patients with VUO: definition and potentially associated factors

We extracted diagnoses as stated in the medical report. Cases without a definite diagnosis or an unweighted differential diagnosis were classified as unknown, and these patients were included in the VUO group. All patient consultations with a determined aetiology for vestibular symptoms were classified in the non-VUO group.

The following variables were considered as potentially associated with visits by patients with VUO: i) demographic data: age < 65 years and sex; ii) comorbidities: hypertension, diabetes, dyslipidaemia, peripheral arterial disease (PAD), cardiac disease, vasculitis, coagulopathies, malignancy, and neurological, psychiatric and ear, nose and throat (ENT) disorders; iii) consultation characteristics: triage category (on a scale from 1: life-threatening to 5: non-urgent), which is routinely performed for all patients by specially trained triage nurses using the Swiss Emergency Triage Scale [16], which is similar to the Manchester Triage System [17]; iv) symptoms and signs: headache, aural fullness, paraesthesia, other symptoms of central nervous system (CNS) disorders, types of triggers for the vestibular symptoms (head movements, physical activity, gait, visual), and improvement at rest.

#### ED resource consumption for diagnostics: definition and potential confounders

The ED’s resource consumption on diagnostics was defined as the sum of physician, nurse, radiology, and laboratory resources measured in TP. In addition to the above-mentioned variables, the triage category was included in the list of potential confounders because an urgent triage category per se might result in a more extensive and costly work-up.

### Statistical analysis

We used Stata® 13.1 (StataCorp, The College Station, Texas, USA) to perform the statistical analyses. For descriptive analysis, proportions were presented as their absolute number accompanied by their relative number. As the normal distribution of most continuous variables was not given, the median together with the interquartile range (IQR, 25th percentile to 75th percentile) was presented to describe their distribution. Wilcoxon rank sum tests and chi-square tests were performed to identify potential clinical continuous and categorical characteristics, respectively, which were associated with ED visits by patients with VUO. All variables that showed at least some evidence (*p* < 0.2) [18, 19] against the null hypothesis of no association with VUO visits, were considered as potential associations and included in the multivariable model. A stepwise backward logistic regression model (*p* < 0.05) including all potential associations identified through univariable analysis was calculated to determine associations with ED visits by patients with VUO. The measure of strength was presented with odds ratios (ORs) and 95% confidence intervals (95% CI).

To study the association of consultations by patients with VUO and ED resource consumption, a stepwise backward linear regression model (p < 0.05, listwise regression) was used that included all the potential confounders mentioned above as well as the triage category. As ED resource consumption was not normally distributed, it was logarithmically transformed for the multivariable analysis. Thus, the exponentiated coefficients of the final model correspond to the geometric mean ratio (GMR) of the non-log-transformed ED resource consumption [20]. We did not adjust for multiple testing.

## Results

**Fig. 1:**
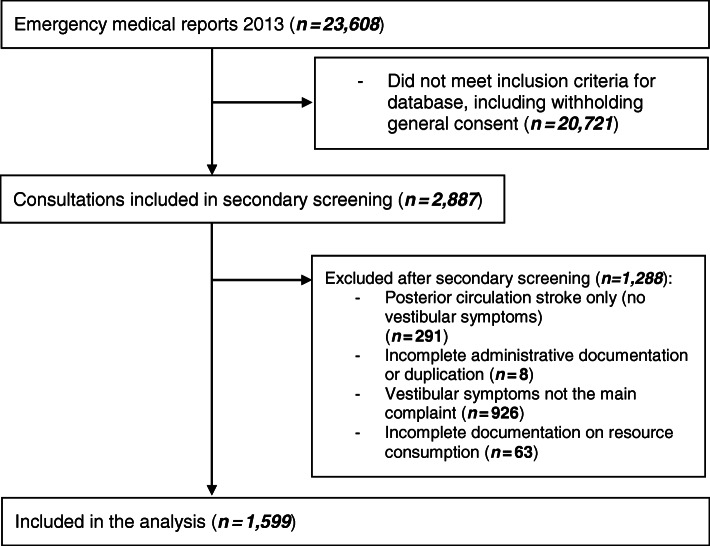
Flowchart of the study

In total, 1599 (6.8%) of the 23,608 ED consultations during the year 2013 were by patients who presented with a main complaint of “vestibular symptoms” and were included in this analysis. The details of the study design and reasons for exclusion are shown in Fig. 1.

The median age was 55 years (IQR 38–69), half of the patients were male (50.5%), and the median triage category was “urgent” (3, IQR: 2–3). Of the consultations, 34.2% were walk-in; 35.3% of all patients were seen by a neurologist and 11.4% were seen by an ENT physician while they were at the ED.

The study population was divided into two groups: consultations with patients in whom the origin of the vestibular symptoms was unknown at discharge (VUO group: *n* = 229, 14.3%), and consultations where the origin of the vestibular symptoms had been determined (non-VUO group: *n* = 1370, 86.7%). Frequently recorded aetiologies for vertigo were as follows (multiple nomination possible): dysautonomy (17.7%; *n* = 243), stroke (14.2%; *n* = 194), peripheral-vestibular (12.7%; *n* = 174), cardiovascular (12.5%; *n* = 171), infection-related (8.0%; *n* = 109), other CNS-related (7.8%; *n* = 107), and drug-related (5.0%; *n* = 68).

### Characteristics that are associated with consultations by patients with vestibular symptoms of unknown origin (VUO)

Associations of various patient characteristics and acute signs and symptoms in the VUO and non-VUO group are shown in Table 1 and Supplement 1.

**Table 1:** Univariable associations of clinical characteristics according to origin of vertigo (VUO vs. non-VUO groups)

	Total (n = 1599)		VUO (*n* = 229)		Non-VUO (n = 1370)		*p*-value
Demographic characteristics							
Age < 65 years (%)	1065	(66.6)	167	(72.9)	898	(65.5)	0.028
Sex, female (%)	790	(49.4)	126	(55.0)	664	(48.5)	0.066
Comorbidity							
Hypertension (%)	665	(41.6)	94	(41.0)	571	(41.7)	0.858
Diabetes (%)	179	(11.2)	18	(7.9)	161	(11.8)	0.084
Dyslipidaemia (%)	364	(22.8)	43	(18.8)	321	(23.4)	0.120
Peripheral artery Disease (%)	40	(2.5)	8	(3.5)	32	(2.3)	0.299
Cardiac disease (%)	474	(29.6)	64	(27.9)	410	(29.9)	0.544
Vasculitis (%)	8	(0.5)	1	(0.4)	7	(0.5)	0.883
Coagulopathy (%)	13	(0.8)	2	(0.9)	11	(0.8)	0.913
Malignancy (%)	99	(6.2)	13	(5.7)	86	(6.3)	0.727
Neurological disorder (%)	502	(31.4)	87	(38.0)	415	(30.3)	0.020
Psychiatric disorder (%)	330	(20.6)	53	(23.1)	277	(20.2)	0.311
ENT disorder (%)	114	(7.1)	18	(7.9)	96	(7.0)	0.642
Signs and symptoms							
Headache (%)	427	(26.7)	70	(30.6)	357	(26.1)	0.153
Aural fullness (%)	20	(1.3)	7	(3.1)	13	(0.9)	0.008
Paraesthesia (%)	197	(12.3)	42	(18.3)	155	(11.3)	0.003
Other neurological symptoms (%)	480	(30.0)	59	(25.8)	421	(30.7)	0.129
Trigger, head Movements (%)	139	(8.7)	28	(12.2)	111	(8.1)	0.040
Trigger, effort (%)	68	(4.3)	6	(2.6)	62	(4.5)	0.186
Trigger, walking (%)	203	(12.7)	37	(16.2)	166	(12.1)	0.089
Trigger, visual (%)	20	(1.3)	6	(2.6)	14	(1.0)	0.044
Improvement at rest (%)	185	(11.6)	39	(17.0)	146	(10.7)	0.005

*Abbreviations*: *ENT* Ear, nose and throat, *VUO* Vestibular symptoms of unknown origin

A logistic multivariable regression with all the potential associations identified through univariable analysis (*p* < 0.2) and stepwise removal of non-significant (*p* > 0.05) variables led to the final model shown in Table 2.

**Table 2:** Patient characteristics associated with a final diagnosis of vestibular symptoms of unknown origin among emergency consultations with patients presenting with vestibular symptoms through multivariable logistic regression analysis (n = 1599)

	OR	95% CI	p-value
**Demographic characteristics characte**			
Age < 65 years	1.39	1.01–1.92	0.042
Neurological comorbidity	1.48	1.10–1.99	0.010
**Symptoms and signs**			
Paraesthesia	1.72	1.17–2.52	0.005
Aural fullness	2.95	1.15–7.60	0.025
Improvement at rest	1.73	1.17–2.55	0.006

*Abbreviations*: *CI* Confidence Interval, *OR* Odds Ratio

Aural fullness showed the strongest association with VUO (OR 2.9, 95% CI: 1.1–7.3, *p* = 0.030). Other characteristics that were associated with VUO were: age younger than 65 years, the presence of a neurological comorbidity, paraesthesia and improvement at rest, with ORs ranging from 1.4–1.8 (Table 2).

Inclusion of all potential associations shown in Table 1 in a logistic regression model with stepwise forward selection of the variables (p < 0.2) revealed the same final model.

### Resource consumption in the ED

The resources consumed during consultations for VUO and non-VUO are shown in Table 3. Consultations with patients with VUO consumed more ED resources (median VUO 1170 TP, IQR 628–1661 vs. non-VUO 831 TP, IQR 523–1552, *p* < 0.001). In the subgroup analysis, the differences in ED resource consumption were also evident in all subgroups of physician work and radiology resources; 47.6% of the VUO consultations included a CT scan (vs. 32.6% non-VUO) and 34.9% an MRI scan (vs. 25.4% non-VUO).

**Table 3:** Emergency department resource consumption in tax points (TP, medical currency) in total and according to the vestibular symptom group (VUO vs. non-VUO)^a^

	Total (n = 1599)		VUO (n = 229)		Non-VUO (n = 1370)		p-value
Physician work (TP)	389	(282–515)	435	(358–562)	382	(273–509)	< 0.001
Physician check-up effort (TP)	195	(115–231)	222	(142–258)	186	(107–231)	< 0.001
Physician admin work (TP)	107	(53–160)	160	(71–213)	107	(53–160)	< 0.001
Physician medical report work (TP)	39	(39–71)	39	(39–71)	39	(39–71)	0.008
Nurse work (TP)	98	(35–98)	62	(35–98)	98	(35–98)	0.119
Laboratory resources (TP)	141	(81–239)	140	(82–226)	141	(81–239)	0.821
Radiology resources (TP)	82	(0–952)	484	(0–952)	67	(0–952)	0.007
Sonography [n (%)]	162	(10.1)	25	(10.9)	137	(10.0)	0.670
X-ray [n (%)]	285	(17.8)	31	(13.5)	254	(18.5)	0.067
CT scan [n (%)]	555	(34.7)	109	(47.6)	446	(32.6)	< 0.001
MRI scan [n (%)]	428	(26.8)	80	(34.9)	348	(25.4)	0.003
Total ED resources (TP)	867	(532–1577)	1170	(628–1661)	831	(523–1552)	< 0.001
Total ED costs (US$)	1382	(920–2044)	1520	(1008–2124)	1356	(911–2022)	0.018

^a^The values are presented as TP with median (interquartile range), unless otherwise specified

*Abbreviations*: *CT* Computed Tomography, *ED* Emergency Department, *MRI* Magnetic Resonance Imaging, *VUO* Vestibular Symptoms of Unknown Origin

The pattern of resource consumption distribution was similar between VUO and non-VUO patients (see Supplement 2). Almost half of the resources were accounted for by physicians’ work (VUO: 46.1% vs. non-VUO: 47.9%), followed by radiology (VUO: 30.6% vs. non-VUO: 24.6%), and laboratory resources (VUO: 15.9% vs. non-VUO: 18.2%).

The multivariable analysis confirmed the positive association between VUO and total consumption of ED resources: the geometric mean of the total ED resources was 1.2 times higher for consultations with VUO patients than for those with non-VUO (GMR: 1.2, 95% CI: 1.1–1.3, p < 0.001). This association was adjusted for the following variables that were considered in the final model (stepwise backward selection, *p* < 0.05): age < 65 years, triage, trigger (visual and head movements), improvement at rest, paraesthesia, and other neurological symptoms, as well as the comorbidities hypertension, dyslipidaemia, and neurological disorder (Supplement 3).

## Discussion

This retrospective cross-sectional study included all emergency consultations with patients whose main complaint was vestibular symptoms presenting at our tertiary ED over one year. Consultations with patients with VUO and their ED resource consumption were studied in detail and compared to non-VUO patients. The main findings are as follows: 1) Age < 65 years, neurological comorbidity, aural fullness and accompanying paraesthesia are associated with VUO. 2) Patients with VUO were comparatively rare in our population, reflecting high diagnostic accuracy and justifying the use of additional ED diagnostic resources, including neuroimaging, if examination does not reveal the underlying problem.

### Characteristics of VUO consultations

In the multivariate regression, we identified several factors that are associated with discharging a patient with vestibular symptoms without a definitive diagnosis. Younger patients were more likely to have VUO. However, several possible aetiologies for vestibular symptoms that are investigated in the ED setting are more common in older patients [21], suggesting that causes of vestibular symptoms may differ between younger and older patients. Also, limited mobility, postural instability and the risk of falls increase with age [22]. Given that cerebro- and cardiovascular risk factors did not differ between VUO and non-VUO patients, this finding is particularly interesting: Vestibular symptoms are common in young patients with missed ischaemic stroke [23]. Risk stratification is commonly used to guide decision-making in the ED, especially if there is a long list of differential diagnoses. Clinical decision rules can help to rule out one particular diagnosis but do not solve the puzzle, as is the case with vestibular symptoms [24]. Given that stroke has been described as a relatively rare cause of vestibular symptoms [2], young patients in particular may be considered as “low-risk” patients.

Patients with a neurological comorbidity and those reporting paraesthesia were more likely to have VUO. Both vestibular symptoms and paraesthesia are relatively nonspecific symptoms of various underlying disorders [25]. A neurological comorbidity is a special challenge for doctors as vestibular symptoms may or may not be associated with the pre-existing disease. Further, acute exacerbations of chronic vestibular symptoms are difficult to distinguish from a new aetiology. As neurocognitive and neuropsychiatric symptoms are common in patients with neurological comorbidities, general internal and neuro-otological evaluation may be more complicated (e.g. due to reduced cooperation), leading to a lower diagnostic accuracy and thus more VUO cases.

The positive association of VUO with aural fullness is suggestive of a hydroptic ear disease (Menière’s disease) [26]. As the rate of diagnosed peripheral-vestibular disorders was comparatively low in our ED [2], and only 11.4% of all patients with VUO were examined by an ENT physician, it is likely that some patients with peripheral-vestibular problems were misclassified as VUO patients. In particular, patients with episodic vestibular symptoms may be oligosymptomatic or asymptomatic at the time of ED presentation, thus complicating the diagnosis. Given the considerable impact vestibular symptoms have on quality of life [27–29], ED physicians should strive to identify possibly treatable aetiologies as early as possible to minimize the likelihood of their becoming chronic. Owing to the retrospective nature of the study, and because there was often no assessment by an ENT physician, it is possible that incomplete hearing loss was missed by physicians and misinterpreted by patients as aural fullness. We would thus encourage physicians to take a careful, detailed and structured history, as acute cochleovestibular hearing loss, which can be identified using the head-impulse, nystagmus, test-of-skew (HINTS)-plus test (HINTS-plus finger-rub hearing test) is also typical of labyrinthine infarctions [30].

### Resource use

Patients with vestibular symptoms are frequently seen in the ED and evaluation has to take into account common, potentially dangerous aetiologies such as strokes [2, 4].

Various studies have investigated ED resource consumption by patients with certain vestibular diagnoses and reported frequent use of imaging [8–11]. However, to our knowledge, resource consumption by patients with VUO has not yet been investigated. Compared to previous findings that 20–30% of patients are discharged without a definite diagnosis, the percentage of patients with VUO in our ED (14.3%) was relatively low [1, 2, 9]. This might be because our ED physicians receive regular training on examination techniques for vestibular symptoms (e.g. HINTS, positional manoeuvres), interdisciplinary diagnosis and treatment. Also, neurologists, neuroradiologists and ENT physicians are present round-the-clock in the ED. While nurse and laboratory costs were similar for VUO and non-VUO patients, physicians’ patient and administration time was significantly higher for VUO patients. This finding is likely to be explained by detailed history taking, thorough clinical examination and frequent consultation with a second specialist. Clinical examination of patients with vestibular symptoms is important to narrow down the list of possible differential diagnoses, confirm suspected clinical diagnoses (e.g. BPPV) and to increase pre-test probability if further diagnostic testing is necessary (e.g. HINTS-plus, which enables the physician to distinguish central from peripheral aetiologies in patients with acute vestibular syndrome with a high sensitivity [30, 31]).

Furthermore, MRI was performed more often in our ED than has previously been reported (i.e. in 34.9% of VUO consultations). Previous studies have reported additional diagnostics with CT in 39.4% and with MRI in only 2.3% of patients [8]. The more frequent use of MRI has increased diagnostic accuracy by enabling life-threatening conditions to be ruled out, and although this is not cost-effective in the short-term it is certainly beneficial from a long-term perspective. Every scan that reveals a serious and treatable underlying cause (e.g. an ischaemic stroke) contributes to reducing morbidity. This presumably outweighs any possible cost reduction that might be obtained by a rigorous limitation of the use of (neuro-)imaging. In the context of vestibular symptoms, choosing the right MRI sequence is of the utmost importance: MRI according to a conventional stroke protocol is less sensitive than a HINTS-plus to detect small strokes causing vestibular symptoms [32]. Where a brainstem infarction is suspected, thin-sliced 3 mm diffusion-weighted imaging (DWI) sequences should be added, as they have a higher sensitivity for the detection of acute and subacute brainstem infarctions [33]. If imaging reveals an underlying pathology, targeted treatment can be initiated to reduce further invalidity. Therefore, we recommend MRI if the aetiology of vestibular symptoms remains unclear despite thorough clinical examination. An inconclusive or “negative” MRI at the ED should never be used as the grounds for ruling out a clinically suspected pathology [32]. The aim of saving costs and time might explain why CT was the most-used imaging technique. Previous studies reported that CT is often performed to exclude cerebrovascular events [8, 9]. However, CT is inferior to MRI for detecting small brain lesions, especially in the posterior fossa [34, 35]. Ammar et al. (2017) reported that CT only led to a diagnosis in 3.6% of all patients, while MRI added diagnostic value in 12%, and half of the patients examined had had a stroke [9]. Replacing MRI with CT to rule out a dangerous diagnosis is an ineffective way of reducing costs given its low sensitivity [9].

Total resource consumption by VUO patients was significantly higher than that by patients whose vestibular symptoms had a determined aetiology. High diagnostic uncertainty leads to additional diagnostic efforts. To determine whether the indication for the tests performed was correct lies beyond the scope of this analysis. However, the proportion of VUO patients was comparatively small in our population, suggesting that additional testing may increase the diagnostic accuracy, allowing targeted treatment (e.g. secondary stroke prevention) and thus improving long-term outcomes [36].

Various groups have attempted to develop algorithms and pathways for ED encounters with patients with vestibular symptoms [31, 37–39]. However, these approaches focus on distinguishing between central and peripheral-vestibular vertigo, mostly defined as rotational vertigo, although the type of vestibular symptoms often does not correlate with the underlying aetiology [40, 41]. Prospective studies should aim at increasing diagnostic accuracy while reducing unnecessary and potentially harmful diagnostic measures. Diagnostic accuracy can be improved by: a) thorough differentiation of timing and triggers [37]; b) performing a thorough clinical examination either helping to confirm a suspected diagnosis (e.g. BPPV) or increasing pre-test probability for further diagnostic measures; and c) developing algorithms that include all types of vestibular symptoms regardless of aetiology, as suggested by the Bárány Society [12]. This allows the possible differential diagnoses to be narrowed down and leads to targeted history taking, clinical examination and (neuro-)imaging. Instruments such as video-oculography devices, and wide implementation of targeted MRI protocols could improve differential diagnosis and should be readily available [42].

### Strengths and limitations of the study

This study has several strengths: It was conducted in an interdisciplinary ED and had broad inclusion criteria. Together with the meticulous screening method and reassessment of unclear cases by neuro-otology adjudicators, this contributed to minimizing selection bias. Such bias is a high risk in studies that include only patients who were seen by a physician in a predefined discipline or when inclusion is based on diagnostic codes. Furthermore, VUO patients were relatively rare in our study compared to other studies, which might be attributable to the high availability of specialists in various disciplines, and of MRI facilities, resulting in high diagnostic accuracy [2]. Finally, this study, albeit cross-sectional, addresses a clinically relevant topic, that has not been investigated so far.

However, our study was conducted in a tertiary care centre and therefore referral bias could have led to a shift of aetiologies towards more severe or rarer disorders. Patients with VUO might not have been referred, and the proportion of VUO might be higher at community hospitals. Also, prevalence of reported symptoms and signs might have been underestimated due to the reporting bias that systematically occurs in retrospective studies based on medical reports. Furthermore, we were not able to enumerate the contribution of each single diagnosis in patients with multiple morbidities whose vestibular symptoms cannot necessarily be attributed to a single aetiology, and are classified as VUO. Given the cross-sectional study design and the lack of follow-up data on underlying causes that were eventually identified, it should be complemented by prospective studies in order to validate the associated factors as predictors for correct triage and management of VUO. Lastly, this is retrospective data that is susceptible to information bias and special examinations such as caloric and hearing tests are reflected by physicians’ work only and not included in the total ED resource consumption although they are reflected by the overall costs.

## Conclusions

Patients with dizziness often undergo cost-intensive work-ups in the ED, reflecting greater efforts to identify the cause. Despite higher total resource use compared to patients in whom the origin of vestibular symptoms can be determined, one in seven patients with vertigo is classified as having VUO. Cost-driving factors associated with consultations with VUO patients were age < 65 years, neurological comorbidity, aural fullness and accompanying paraesthesia.

Careful history taking, targeted clinical examination and additional exams (e.g. MRI) in selected patients play a key role in the cost-efficient work-up of patients with vestibular symptoms.

## Supplementary information

**Additional file 1: Supplement 1.** Univariable associations of clinical characteristics according to origin of vertigo (VUO vs. non-VUO visits), *n* = 1599.

**Additional file 2: Supplement 2.** Relative resource consumption distribution in VUO (*n* = 229) and non-VUO (*n* = 1370) consultations.

**Additional file 3: Supplement 3.** Multivariable linear regression to determine the strength of association of patient characteristics with the total ED resource consumption in VUO compared to non-VUO consultations (*n* = 1562^a^).

## Data Availability

The statistical data used to support the findings of this study are available from the corresponding author upon request.

## Abbreviations

BPPV: Benign paroxysmal positional vertigo
CNS: Central nervous system
DWI: Diffusion-weighted imaging
ED: Emergency department
ENT: Ear, nose and throat
GMR: Geometric mean ratio
HINTS: Head-impulse, nystagmus, test-of-skew
OR: Odds ratio
PAD: Peripheral arterial disease
TP: Tax points
VUO: Vestibular symptoms of unknown origin
