# Supplementary material for: Characteristics and resource needs in patients with vestibular symptoms: a comparison of patients with symptoms of unknown versus determined origin
Source: BMC Emerg Med. 2020 Aug 31;20:70. doi: 10.1186/s12873-020-00361-8 (PMC7460761; doi:10.1186/s12873-020-00361-8)
Supplement: Supplementary file 1 — Additional file 1: Supplement 1. Univariable associations of clinical characteristics according to origin of vertigo (VUO vs. non-VUO visits), n = 1599. [file 12873_2020_361_MOESM1_ESM.docx]

### Supplement 1: Univariable associations of clinical characteristics according to origin of vertigo (VUO vs. non-VUO visits), n=1,599

| **VUO vs. non-VUO consultations** | **OR** | **95% CI** | **p-value** |
| --- | --- | --- | --- |
| **Demographic characteristics** |  |  |  |
| Age <65 years | 1.42 | 1.04–1.93 | 0.029 |
| Sex, female | 1.30 | 0.98–1.72 | 0.067 |
| **Comorbidity** |  |  |  |
| Hypertension | 0.97 | 0.73–1.29 | 0.858 |
| Diabetes | 0.64 | 0.39–1.07 | 0.086 |
| Dyslipidaemia | 0.76 | 0.53–1.08 | 0.121 |
| PAD | 1.51 | 0.69–3.33 | 0.302 |
| Cardiac disease | 0.91 | 0.67–1.24 | 0.544 |
| Vasculitis | 0.85 | 0.1–6.97 | 0.883 |
| Coagulopathy | 1.09 | 0.24–4.94 | 0.913 |
| Malignancy | 0.9 | 0.49–1.64 | 0.727 |
| Neurological comorbidity | 1.41 | 1.05–1.89 | 0.021 |
| Psychiatric comorbidity | 1.19 | 0.85–1.66 | 0.312 |
| ENT comorbidity | 1.13 | 0.67–1.91 | 0.643 |
| **Symptoms and signs** |  |  |  |
| Headache | 1.25 | 0.92–1.69 | 0.154 |
| Aural fullness | 3.29 | 1.30–8.34 | 0.012 |
| Paraesthesia | 1.76 | 1.21–2.56 | 0.003 |
| Other neurological symptom | 0.78 | 0.57–1.07 | 0.130 |
| Trigger, head movements | 1.58 | 1.02–2.45 | 0.042 |
| Trigger, effort | 0.57 | 0.24–1.33 | 0.192 |
| Trigger, walking | 1.4 | 0.95–2.06 | 0.090 |
| Trigger, visual | 2.61 | 0.99–6.85 | 0.052 |
| Improvement at rest | 1.72 | 1.17–2.53 | 0.006 |

**Abbreviations:** ENT, ear, nose and throat; PAD, Peripheral arterial disease; VUO, Vestibular symptoms of unknown origin.
