# Supplementary material for: Characteristics and resource needs in patients with vestibular symptoms: a comparison of patients with symptoms of unknown versus determined origin
Source: BMC Emerg Med. 2020 Aug 31;20:70. doi: 10.1186/s12873-020-00361-8 (PMC7460761; doi:10.1186/s12873-020-00361-8)
Supplement: Supplementary file 2 — Additional file 2: Supplement 2. Relative resource consumption distribution in VUO (n = 229) and non-VUO (n = 1370) consultations. [file 12873_2020_361_MOESM2_ESM.docx]

### Supplement 2: Relative resource consumption distribution in VUO (n=229) and non-VUO (n=1,370) consultations

**Abbreviations:** VUO, Vestibular symptoms of unknown origin.
