# Supplementary material for: Characteristics and resource needs in patients with vestibular symptoms: a comparison of patients with symptoms of unknown versus determined origin
Source: BMC Emerg Med. 2020 Aug 31;20:70. doi: 10.1186/s12873-020-00361-8 (PMC7460761; doi:10.1186/s12873-020-00361-8)
Supplement: Supplementary file 3 — Additional file 3: Supplement 3. Multivariable linear regression to determine the strength of association of patient characteristics with the total ED resource consumption in VUO compared to non-VUO consultations (n = 1562a). [file 12873_2020_361_MOESM3_ESM.docx]

### Supplement 3. Multivariable linear regression to determine the strength of association of patient characteristics with the total ED resource consumption in VUO compared to non-VUO consultations (n=1,562^a^)

|  | **GMR** | **95% CI** | **p-value** |
| --- | --- | --- | --- |
| **VUO** | 1.22 | 1.11–1.34 | <0.001 |
|  |  |  |  |
| **Adjusted for…** |  |  |  |
| Age <65 years | 0.89 | 0.82–0.96 | 0.004 |
| Triage less acute (per category)^b^ | 0.80 | 0.76–0.84 | <0.001 |
| Hypertension | 1.14 | 1.05–1.24 | 0.001 |
| Dyslipidaemia | 1.11 | 1.02–1.21 | 0.017 |
| Neurological comorbidity | 1.12 | 1.05–1.21 | 0.002 |
| Headache | 1.20 | 1.11–1.29 | <0.001 |
| Trigger, visual | 1.35 | 1.01–1.82 | 0.043 |
| Trigger, head movements | 0.86 | 0.76–0.98 | 0.021 |
| Improvement at rest | 1.19 | 1.07–1.33 | 0.002 |
| Paraesthesia | 1.21 | 1.1–1.34 | <0.001 |
| Other central symptoms | 1.24 | 1.15–1.33 | <0.001 |

^a^ 37 consultations (2.3%) were excluded because the triage category was not known.

^b^ From one triage category, to the next less acute triage category.

**Abbreviations:** ED, Emergency Department; GMR, Geometric Mean Ratio; VUO, Vestibular symptoms of unknown origin.
